# Effects of Tartary Buckwheat Protein on Gut Microbiome and Plasma Metabolite in Rats with High-Fat Diet

**DOI:** 10.3390/foods10102457

**Published:** 2021-10-15

**Authors:** Jing Liu, Yu Song, Qi Zhao, Yuguo Wang, Congshou Li, Liang Zou, Yichen Hu

**Affiliations:** Key Laboratory of Coarse Cereal Processing, Ministry of Agriculture and Rural Affairs, Sichuan Engineering & Technology Research Center of Coarse Cereal Industralization, School of Food and Biological Engineering, Chengdu University, Chengdu 610106, China; liujing2120@126.com (J.L.); songyu@cdu.edu.cn (Y.S.); zhaoqi@cdu.edu.cn (Q.Z.); wyg19822928354@126.com (Y.W.); lcs17687189361@126.com (C.L.); zouliang@cdu.edu.cn (L.Z.)

**Keywords:** Tartary buckwheat protein, hyperlipidemia, gut microbiota, metabolomics

## Abstract

The prevalence of lipid metabolism diseases, mainly obesity, fatty liver, and hyperlipidemia, is increasing in the world. Tartary buckwheat is a kind of medicinal and edible crop, and clinical experiments have also confirmed that dietary Tartary buckwheat can effectively regulate lipid metabolism disorders. Tartary buckwheat protein (TBP), as the main active ingredient of Tartary buckwheat, has an effect of blood lipid reduction that has been widely reported. In this paper, we investigated the constituents of TBP and then evaluated the hypolipidemic effect of TBP in hyperlipidemia rats. Male Sprague–Dawley rats were fed a high-fat diet for six weeks to induce hyperlipidemia and then given TBP orally for five weeks. The effects of TBP on body weight, serum lipids, liver lipids, liver oxidative stress, pathological organization, gut microbiota, and plasma metabolites were analyzed. At the serum level, TBP supplement significantly decrease the level of LDL-C and increase the level of HDL-C. At the liver level, it can reduce the levels of TC, TG, and LDL-C. The potential mechanism of action is, on the one hand, to increase the abundance of the Lachnospiraceae and the Ruminococcaceae by modulating the gut microbiota, facilitating the productivity of short-chain fatty acids, and increasing fecal bile acid excretion and, on the other hand, may be related to the improvement of bile acid metabolism.

## 1. Introduction

In the last few decades, changes in lifestyles and unhealthy eating patterns of people have culminated in an increasing incidence of cardiovascular and cerebrovascular diseases [1]. Among them, the proportion of high-calorie diets in the dietary structure is increasing, and long-term high-fat diet (HFD) may result in an imbalance of energy intake and energy expenditure and ultimately may lead to lipid metabolism disorder [2]. Features of lipid metabolism disorder are elevated lipid concentrations in the blood and liver, and long-term ingestion of HFD can induce intense oxidative stress [3]. Furthermore, lipid metabolism disorder is linked to an increased risk of numerous diseases, including obesity, hyperlipidemia, atherosclerosis, fatty liver, and heart disease, which reduce the people’s quality of life. The International Diabetes Federation is of the view that a quarter of adults worldwide suffer from lipid metabolism disorder [4]. Thus, the rising incidence rate of lipid metabolism disorder has grown to be a serious public health concern.

To alleviate lipid metabolism disorder, pharmacological treatment has been applied. Currently, the commonly used lipid-lowering drugs have made considerable progress, but the negative side effects of these medications have increased over time. For instance, statins, which structure analogs to hydroxymethylglutaryl-CoA, are toxic to multiple organs of the human body [5,6]. In addition, long-term use of such medicines may lead to liver cell damage, hepatitis, gastrointestinal disorders, and impaired body resistance to a certain extent [7]. Growing body of literature reports that changes in diet, which involve the use of some foods of plant origin, provide an efficacious treatment to alleviate lipid metabolism disorders and its complications [8]. Thus, the demand from consumers for a healthier diet through naturally occurring, pro-health products continue to grow. It is extremely necessary to search for innovative, naturally occurring ingredients for lipid-reducing foods.

Pseudo cereal is abundant in nutritional compounds, which have been correlated with improved weight loss, antioxidants, and lipid metabolism in individuals fed a dietary supplement enriched with whole grains [9]. Tartary buckwheat (*Fagopyrum tataricum* L., Gaertn.) is a pseudo cereal that primarily thrives in Asia [10]. There is long-term tradition of using Tartary buckwheat flour as either a functional food or a regular diet for the therapy of cardiovascular disease and diabetes in China [11]. Tartary buckwheat protein (TBP), as the main active ingredient of Tartary buckwheat, has been widely reported on regarding the prevention of hyperlipidemia [12,13,14]. Studies have shown that [13], compared with rice protein and wheat protein, adding TBP to the diet can significantly reduce hamster plasma total cholesterol (TC), which mainly through upregulation of hepatic CYP7A1 facilitates the bile acid excretion and also through downregulation of NPC1L1, ACAT2, and ABCG5/8 in the intestine inhibits the resorption of dietary cholesterol. X.L. Zhou et al. [14] also found that dietary TBP can reduce plasma total cholesterol and triglyceride levels in HFD C57BL/6 mice and investigated the influence on gut microbiota. However, they only quantitatively analyzed six species of bacteria in the feces of C57BL/6 mice, including Bifidobacterium, Lactobacillus, Enterococcus, Escherichia, Bacaeroides, and Clostridium, which was not comprehensive and in-depth. In addition, there are not any studies on TBP for metabolic regulatory mechanisms.

Evidence is mounting that the gut microbiota contributes to the progression of obesity [15]. Many literatures have confirmed that alterations in the gut microbiota will affect the metabolism of the host, including vascular function, lipid metabolism, metabolism, and inflammation [16,17,18], indicating that the gut microbiota may be considered to be related to the prophylaxis or therapy of both obesity and related persistent diseases. In addition, metabolomics is also regularly used for lipid metabolism studies. Recent studies have found that the distribution of plasma metabolites in obese patients has undergone significant changes [19].

Thus, in this study, experimental material TBP was firstly analyzed in experiments to investigate the protein composition of TBP. To assess the effect of TBP on lipid levels in hyperlipidemic rats, the levels of serum and liver lipid profiles of rats in different treatment groups were tested, and histopathological evaluation of liver was performed. To assess the effect of TBP on hepatic oxidative stress in hyperlipidemic rats, the levels of hepatic oxidative factors in rats from different treatment groups were tested. In addition, to probe the mechanism of action of TBP, the levels of rat fecal short-chain fatty acids (SCFAs) were measured, and the composition and abundance of the gut microbiota were explored using 16s rDNA high-throughput sequencing. Finally, the effect of TBP on plasma metabolism in hyperlipidemic rats was also assessed.

## 2. Materials and Methods

### 2.1. Materials

Tartary buckwheat seeds were acquired from the Key Laboratory of Coarse Cereals Processing, Ministry of Agriculture and Rural Affairs, Chengdu University (Chengdu, China). Male Sprague–Dawley rats were purchased from the Chengdu Dashuo Experimental Animals Co., Ltd. (Chengdu, China). The total cholesterol (T-CHO), triglycerides (TG), high-density lipoprotein cholesterol (HDL-C), low-density lipoprotein cholesterol (LDL-C), alpha-alanine aminotransferase (ALT), aspartate aminotransferase (AST), free fatty acid (FFA), catalase (CAT), reduced glutathione (GSH), superoxide dismutase (SOD), malondialdehyde (MDA), total antioxidant capacity (T-AOC), and total bile acid (TBA) kits were purchased from Nanjing Jiancheng Bioengineering Institute (Nanjing, China). All other chemicals and reagents are analytical grade.

### 2.2. Preparation of TBP

The extraction method of TBP was according to the method of Guo et al. [20]. Skimming of Tartary buckwheat flour using n-hexane under successive stirring for 24 h. The defatted Tartary buckwheat powder was air-dried and manually mixed into phosphate buffer (pH 7.2, 10 mmol/L) in the proportion of 1:10 (*w*/*v*). It was stirred for 60 min and then centrifuged (5000× *g* for 20 min). Then, we precipitated the supernatant with solid ammonium sulfate at 70–95% saturation. After centrifugation (10,000× *g* 4 °C for 20 min), the sediment was resolved again in buffer, dialyzed (4 °C, 3500 Da), and freeze-dried.

### 2.3. SDS-PAGE and NanoElute-UHPLC-Tims TOF/MS Analysis of TBP

SDS-PAGE experiments were performed using the method of Zhou et al. [21].

Powder lysis buffer (8 M urea (Sigma-Aldrich, Saint Louis, MO, USA), 1% protease inhibitor (Merck Millipore)) was added to TBP samples, lysed by ultrasonication, and centrifuged (4 °C, 12,000× *g*, 10 min), and the protein concentration in the supernatant was determined using a BCA kit (Beyotime Biotechnology, Shanghai, China). Trichloroacetic acid (TCA) was slowly added to the supernatant. After 2 h, the sediment was laundered with pre-chilled acetone. The precipitate was air-dried and added to 200 mM TEAB and sonicated, and trypsin was added at a proportion of 1:50 (protease: protein, m/m) and digested overnight. The peptides were solubilized by using mobile phase A. An aqueous solution containing 0.1% formic acid and 2% acetonitrile constitutes mobile phase A, and a solution containing 0.1% formic acid and acetonitrile constitutes mobile phase B. The liquid phase gradient settings were: 0–70 min, 6–24% B; 70–84 min, 24–35% B; 84–87 min, 35–80% B; and 87–90 min, 80% B and the flow rate kept at 450 nL/min. With separation on a NanoElute UHPLC system (Bruker Daltonics, Bremen, Germany) and a Bruker Daltonics C18 column (1.9 μm, 75 μm × 100 mm), the peptides were ionized by capillary ion source. Moreover, it was analyzed by timsTOF Pro mass spectrometry (Bruker Daltonics, Bremen, Germany) for the peptide parent ions and their secondary fragments. The TIMS accumulation time and separation duration were respectively fixed at 100 ms. Mobility values range from 0.65–1.3 Vs/cm^2^ (1/K0). The ion transport capillary temperature was 180 °C. At a voltage setting of 1.75 kV for the ion source, with a range of 100–1700 *m*/*z* for the secondary mass spectrometry scan. A parallel accumulation-serial fragmentation (PASEF) mode was used for the data acquisition mode. An acquisition of mass spectra was followed by 10 secondary spectral acquisitions in PASEF mode with parent ion charge numbers between 0 and 5.

### 2.4. Animals, Diets, and Experimental Design

**Figure 1:**
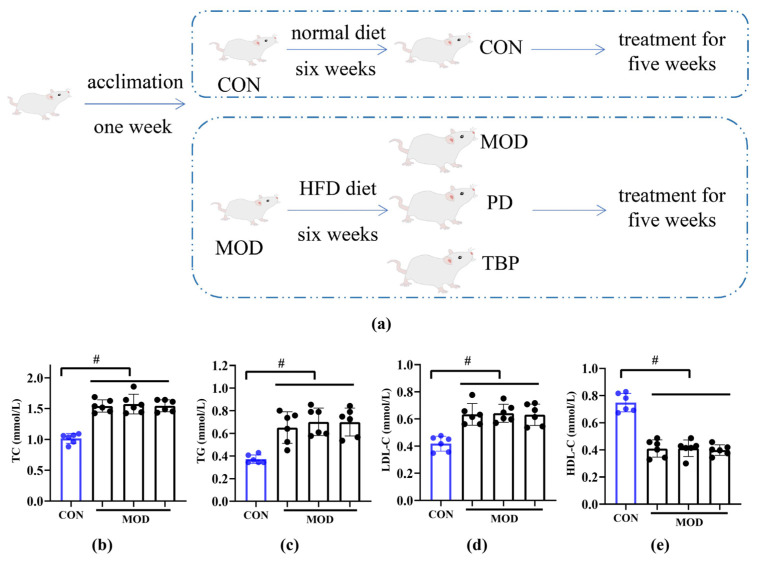
(**a**) The design of the experimental groups. TC (**b**), TG (**c**), LDL-C (**d**), and HDL-C (**e**) in rat serum under a normal diet (CON) and a HFD (MOD) for six weeks. Values are expressed as the mean ± SD (*n* = 6) for all groups. ^#^ *p* < 0.05 denotes statistically significant differences between the CON and MOD groups.

All animal experiments were performed according to protocols approved by the experimental animal ethics committee of Chengdu University. Twenty-four male, pathogen-free Sprague–Dawley (SD) rats (6 weeks old, 180 ± 10 g) were placed in a facility kept at a constant temperature (22 ± 1 °C) and humidity (50 ± 10%) with 12 h light/12 h dark cycle. The animal experiment grouping is shown in Figure 1a. After a seven-day acclimation period, a random division of the rats into four groups was performed (*n* = 6). Besides the control (CON) group (rats fed normal diet), three other groups (model group, MOD) were fed a HFD (consisting of basic feed (63.6%), cholesterol (1.2%), pig fat (15%), sucrose (20%), and bile salt (0.2%)). After six weeks, the rat’s serum TC, TG, LDL-C, and HDL-C levels were measured to assess the establishment of hyperlipidemic rats. There was a statistically significant increase in serum levels of TC (*p* < 0.05), TG (*p* < 0.05), and LDL-C (*p* < 0.05) in the MOD group compared to the CON group, while the levels of HDL-C were decreased significantly (*p* < 0.05), as shown in Figure 1b–e. The experimental results suggest that the hyperlipidemic rat model was successfully prepared, which can be utilized for further experiments. Hyperlipidemic rats were randomly grouped and subdivided by body weight into three groups (*n* = 6): the model (MOD) group, the positive drug (PD) group (selected from the internationally recognized lipid-lowering drug simvastatin), and the Tartary buckwheat protein (TBP) group. Basal diet and HFD diet with 2 mL of 0.9% saline were provided daily in the CON and MOD groups, respectively. A total of 2 mL of simvastatin (1.8 mg/kg·day) was administered orally, and HFD diet was fed in the PD group, while 2 mL of TBP (500 mg/kg·day) was administered orally, and HFD diet was fed in the TBP group. Body weight was measured weekly. Rats were fasted overnight after five weeks of treatment, anesthetized with 2% (*v*/*v*) sodium pentobarbital (0.3 mL/100 g), and then blood samples were collected from the abdominal aorta. Serum and plasma were collected (EDTA was used as an anticoagulant). The cecal feces were collected and quick-frozen with liquid nitrogen for intestinal microbial analysis. In addition, the liver, abdominal fat, and epididymal fat of rats were collected and weighed. Hepatic right lobe and epididymal fat were fixed in 4% paraformaldehyde for HE staining, and the remaining parts were quickly frozen in liquid nitrogen and stored at −80 °C. We calculated the liver index (liver weight(g)/body weight (g)×100%) and abdominal fat rate (abdominal fat tissue weight (g)/body weight (g)×100%) at the end of the study.

### 2.5. Histopathological Analysis

Paraffin-fixed liver and epididymal adipose tissue in 4% paraformaldehyde solution were embedded in paraffin and then sliced for hematoxylin/eosin (HE) staining. Finally, microscopically, the cytoarchitecture of the liver tissue and the cell size of the adipose tissue were observed.

### 2.6. Biochemical Analysis in Serum and the Liver

#### 2.6.1. Lipid Level in Serum

Serum levels of TG, TC, LDL-C, and HDL-C were determined following the manufacturer’s instructions using commercially available kits, and optical density (OD) values were read with a microplate reader (BioTek Instrument, Inc., Winooski, VT, USA). Calculate arteriosclerosis index (AI), AI = (TC − HDL-C)/HDL-C.

#### 2.6.2. Lipid Level in Liver

Determination of TG, TC, LDL-C, and FFA levels in liver tissue was performed following the manufacturer’s instructions using commercially available colorimetric kits.

### 2.7. Liver Function Evaluation

#### 2.7.1. Detection Index of Liver Injury

Measurements of AST and ALT in serum were made with commercially available kits following the manufacturer’s instructions.

#### 2.7.2. Oxidative Stress Marker Analysis in Liver Tissues

Determination of CAT, MDA, GSH, T-AOC levels, and SOD activity in liver tissue was performed with commercially available colorimetric kits based on the manufacturer’s instructions.

#### 2.7.3. Detection of Protein Carbonylation in Rat Liver by Western Blotting

Western blotting was carried out to determine the level of protein carbonylation in rat liver. Experiments were performed by referring to literature methods [22] with minor modifications. Liver tissue was made into 10% liver homogenate using ice in HEPES buffer, and nucleic acids were precipitated using streptomycin sulfate solution. Then, the carbonyl group was specifically derivatized using 2,4-Dinitrophenylhydrazine (DNPH) and precipitated using TCA, among other steps. Protein concentration was measured using the BCA protein quantification kit. Anti-DNP antibody produced in rabbit (D9656, Merck Millipore) was used as primary antibody (dilution: 1:1000), which was subsequently detected by secondary antibody conjugated with horseradish peroxidase (HRP) (dilution: 1:1000) and a chemiluminescent substrate. Up-sampling analysis was performed using the Wes fully automated protein expression subsystem (Protein Simple, San Jose, CA, USA) following the manufacturer’s protocol.

### 2.8. Fecal Analysis

Fecal samples were gathered for one day before the experiment ended, freeze-dried, and kept at −80 °C until being analyzed.

#### 2.8.1. Fecal TC and TBA Analysis

Fecal TC was abstracted with chloroform and methanol. Fecal TBA was abstracted with ethanol. Fecal TC and TBA concentrations were analyzed with commercial kits (Nanjing Jiancheng Bioengineering Institute).

#### 2.8.2. Fecal Cecal SCFAs Measurement

Measurement procedure was based on the literature reported by previous authors [23,24] with some modifications. Briefly, with 4-methylvaleric acid as an internal standard, we added 100 μL 10% H3PO4 and 500 μL methyl tert-butyl ether (containing 50 μg/mL 4-methylvaleric acid) to a 100-mg stool sample and homogenized for 1 min. After centrifugation (12,000× *g*, 4 °C, 10 min), the supernatant was gathered, and a 7000D gas chromatograph-mass spectrometer (Agilent Technologies Inc., Santa Clara, CA, USA) was utilized to characterize and quantify the SCFAs on a HP-INNOWax capillary column (60 m × 0.25 mm × 0.25 μm, Agilent Technologies Inc.). The split ratio was adjusted to 10:1, and the injection volume was 1 μL, 250 °C for the injector, 230 °C for the ion source, and 250 °C for the transmission line. The following was the temperature routine: the initial temperature was 80 °C and then was held there for 3 min, raised to 180 °C at 10 °C/min, raised to 250 °C at 25 °C/min, and then held there for 3 min. The flow of helium was set at 1.0 mL/min.

### 2.9. Cecal DNA Extraction and Sequencing

DNA was obtained from cecum contents using the PowerSoil^®^ DNA Isolation kit following the manufacturer’s instructions. We used the extracted DNA as a template, the 27F forward primer (5′-AGRGTTTGATYNTGGCTCAG-3′), and the 1942R reverse primer (5′-TASGGHTACCTTGTTASGACTT-3′) to amplify the 16S rDNA gene. The PCR analysis was performed on a PCR system (Applied Biosystems 9902). The following PCR conditions were applied: 5 min at 95 °C, followed by 30 cycles of 30 s at 95 °C, 30 s at 50 °C, and finally exothermic at 72 °C for 7 min. There were 5–50 ng of genomic DNA per reaction mixture, KOD FX Neo Buf (2×), 0.8 mM dNTPs (deoxynucleoside triphosphate), and KOD FX Neo (Toyobo). The PCR products were mixed with the loading buffer and then sampled onto agarose gels for detection and quantification using Image J based on the electrophoresis results. After quantification, each sample was mixed in 3 parallels according to the amount of data and fragment size required for each sample. Finally, the beads were recovered and purified using 0.8× magnetic beads (MagicPure Size Selection DNA Beads).

The products from different samples were sequenced on the MiSeq Illumina Sequencing Platform. All high-quality sequencing reads were clustered using USEARCH (version 10.0) with 97% similarity, and operational taxonomic units (OTU) at a certain threshold (0.005%) were acquired. Alpha diversity included the Simpson and Shannon indexes, and beta diversity was visualized by weighted unifrac distance-based non-metric multi-dimensional scaling (NMDS). Similarity analysis was performed to examine the differences in the gut microbiota communities between groups.

### 2.10. UPLC-MS/MS-Based Metabonomics

Plasma samples were prepared by protein precipitation. A total of 600 μL acetonitrile was added to 200 μL serum, vortexed (5 min), and centrifuged (12,000 g/min, 10 min), and the supernatant was collected for analysis.

UPLC analysis was conducted on a Thermo Scientific Vanquish UPLC system (Thermo Fisher Scientific, Waltham, MA, USA). The samples were separated on a Hypersil Gold VANQUISH C18 column (1.8 μm, 100 mm × 2.1 mm, Thermo Fisher Scientific, Waltham, MA, USA). The mobile phase consisted of 0.1% formic acid in acetonitrile (A) and 0.1% formic acid in water (B) at the flow rate of 0.3 mL/min. The gradient elution program was as follows: 0.0–25 min, 5% A to 100% A, then kept at 100% A for 5 min. The samples were maintained in the injection chamber at 4 °C. For each test, 1 μL of plasma sample solution was injected.

Mass spectrometry measurements were performed with a Q-Exactive Focus mass spectrometer (Thermo Fisher Scientific, Waltham, MA, USA). The scanning mode ranged from the full MS to the dd-MS^2^. The scan range of full MS was 50 to 1000 *m*/*z*, and the resolution was 70,000. The resolution of dd-MS^2^ was 17,500, and the (N) CE/stepped nce was 20, 40, and 60. The sheath gas-flow rate was set to 5, and the spray voltage was adjusted to 3.2 kV. The capillary temperature was set to 300 °C, and the S-lens RF level was set to 50.

### 2.11. Statistical Analysis

All results are expressed as mean ± the standard deviation (SD). The differences among the experimental groups were evaluated using one-way analysis of variance (ANOVA), and significant differences among the means were determined using Duncan’s multiple range tests. *p*-Values less than 0.05 were considered statistically significant.

## 3. Results

### 3.1. SDS-PAGE and NanoElute-UHPLC-Tims TOF/MS Analysis of TBP

**Figure 2:**
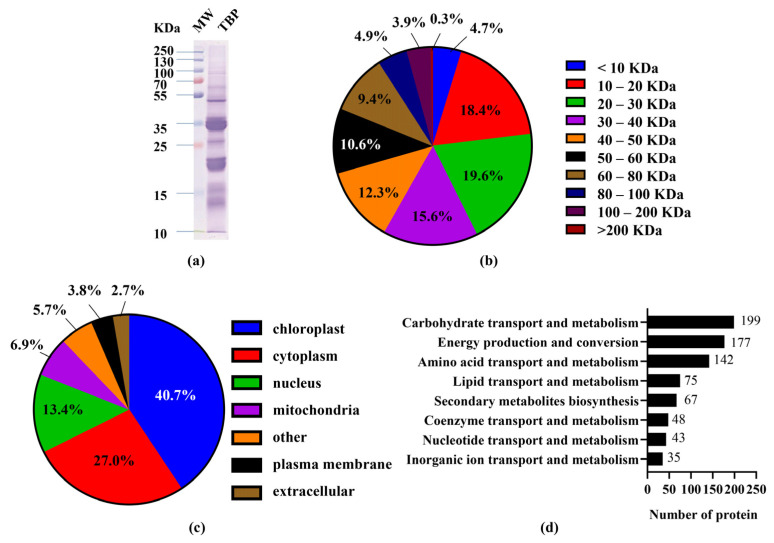
(**a**) SDS-PAGE of TBP; MW, molecular weight marker. (**b**) Molecular weight distribution of the identified proteins in TBP. (**c**) Distribution of the identified proteins in the organelles of TBP. (**d**) Metabolism-related functions of the identified proteins in TBP based on COG/KOG functional classification.

The secondary mass spectrometry data of this experiment were searched with Maxquant (v1.6.15.0, Max Planck Institute of Biochemistry, Munich, Germany), searching the library as Fagopyrum_tataricum_62330_TX_W8084LI_20201202.fasta (31620 sequences), and added to the reverse library to compute the false-positive rate (FDR) due to stochastic matches. In addition, common contamination libraries were added to the database. The accuracy FDR was set at 1% for identification at three levels: spectra, peptides, and proteins, and the identified proteins needed to contain at least one specific (unique) peptide. Altogether, 2310 proteins were characterized in the TBP sampling. The SDS-PAGE maps and protein molecular weight distribution of TBP are shown in Figure 2a,b. A total of 76.6% of the proteins were distributed between 10 and 60 KDa, with less distribution of small and high molecular weight proteins. Subcellular structure annotation of the identified TBP was performed using WolF Psort software. The results are shown in Figure 2c. TBP was mainly distributed in organelles, such as chloroplast, cytoplasm, and nucleus, which is in agreement with the finding of Wang et al. [25]. COG/KOG functional classification statistics were performed based on the EggNOG database (version 5, European Molecular Biology Laboratory (EMBL), Heidelberg, Germany). Among the identified TBPs, 1839 proteins were functionally annotated, included three aspects of information storage and processing, metabolism, cellular processes, and signaling. Among them, number and category description of metabolism-related proteins are shown in Figure 2d, including carbohydrate transport and metabolism, energy production and conversion, amino acid transport and metabolism, lipid transport and metabolism, secondary metabolites biosynthesis, coenzyme transport and metabolism, nucleotide transport and metabolism, and inorganic ion transport and metabolism. Through COG/KOG functional classification, TBP was found to be rich in proteins related to nutrition and metabolism, which can be used as potential molecules for health food or functional food.

### 3.2. Effect of TBP on Body Weight, Liver Index, Abdominal Fat Rate, and Histopathological of HFD-Fed Rats

**Figure 3:**
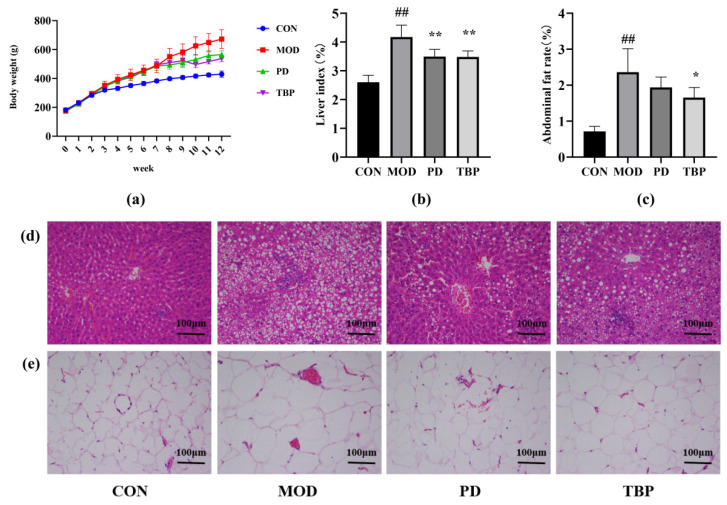
Effect of TBP on body weight (**a**), liver index (**b**), and abdominal fat rate (**c**) in rats consuming a HFD. Histopathological examinations of liver (**d**) and epididymal adipose (**e**) tissues using HE staining (200×). Values are expressed as the mean ± SD (*n* = 6) for all groups. ^##^ *p* < 0.01 denotes statistically significant differences between the CON and MOD groups. ** *p* < 0.01 and * *p* < 0.05 denote statistically significant differences with MOD group.

As Figure 3a shows, the body weight of the rats in each group gradually increased over time, while the rats that received HFD feeding had markedly higher body weight than those on a normal diet. TBP could decrease the body weight of HFD-fed rats, similar to the PD rats. Besides, after being HFD fed for 11 weeks, the liver index of MOD rats was significantly increased compared with CON rats (Figure 3b; *p* < 0.01). For PD rats and TBP rats, liver index was significantly reduced compared with MOD rats (*p* < 0.01). Moreover, MOD rats had much higher abdominal fat rate than CON rats (Figure 3c; *p* < 0.01), while TBP reduced the abdominal fat rate of HFD rats (*p* < 0.05).

To ascertain the ameliorative efficacy of TBP on the histopathological lesions in the liver and epididymal adipose tissues in rats with hyperlipidemia induced by HFD feeding, HE staining experiments were performed. The results are presented in Figure 3d,e. In the CON group, normal liver architecture was observed, while liver tissue injuries, including vacuolization of hepatocytes, severe steatosis, and heavy hepatocyte lipid droplet accumulation, were seen in the MOD rats. However, the PD group attenuated the pathological lesions in hepatic tissues; in particular, no significant lipid droplets were seen in the hepatocytes. Similar to the effects of PD, TBP also improved lipid droplet accumulation in hepatocytes of HFD-fed rats. Additionally, compared with the CON group, adipocytes in the epididymal adipose tissue of rats in the MOD group were significantly enlarged, while the PD and TBP groups achieved partial reversal, indicating that their lipid stores were inhibited.

### 3.3. Effect of TBP on Lipid Levels in Serum and Liver Tissues

**Figure 4:**
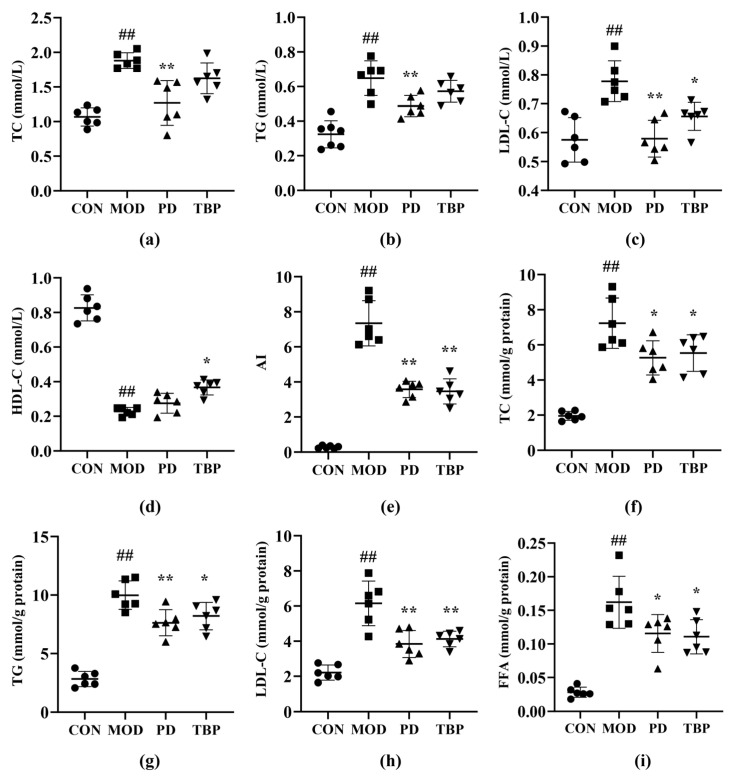
Effect of TBP on serum TC (**a**), serum TG (**b**), serum LDL-C (**c**), serum HDL-C (**d**), AI (**e**), liver TC (**f**), liver TG (**g**), liver LDL-C (**h**), and liver FFA (**i**) in rats consuming a HFD. Values are expressed as the mean ± SD (*n* = 6) for all groups. ^##^ *p* < 0.01 denotes statistically significant differences between the CON and MOD groups. ** *p* < 0.01 and * *p* < 0.05 denote statistically significant differences with MOD group.

As can be seen from Figure 4a–d, at the serum level, the rats in PD group showed no significant difference (*p* > 0.05) in HDL-C, whereas TC, TG, and LDL-C levels reduced remarkably (all *p* < 0.01) as compared to MOD. Serum HDL-C was increased (*p* < 0.05), and serum LDL-C was reduced significantly (*p* < 0.05) in the TBP group compared to that in the MOD group. However, no noticeable difference was observed in TC and TG. Furthermore, the results also indicated that PD and TBP could ameliorate the increased arteriosclerosis index of HFD rats (Figure 4e, *p* < 0.01). At the liver level, MOD group rats showed higher TG, TC, LDL-C, and FFA levels than CON group rats (Figure 4f–i, all *p* < 0.01). Treatments with PD and TBP significantly decreased TG, TC, LDL-C, and FFA levels in liver (all *p* < 0.05). On the basis of these findings, one can conclude that TBP has a positive role in the potential regulation of serum and liver lipids.

### 3.4. Liver Function Evaluation

**Figure 5:**
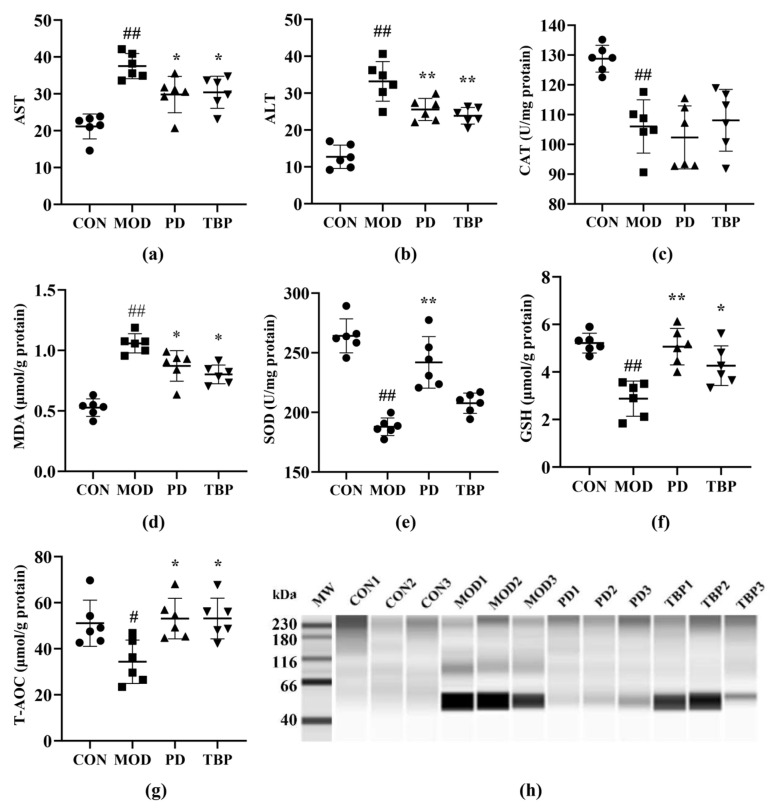
Effect of TBP on serum AST (**a**), serum ALT (**b**), liver CAT (**c**), liver MDA (**d**), liver SOD (**e**), liver GSH (**f**), and liver tT-AOC (**g**) in rats fed a HFD. (**h**) Oxidation levels of carbonyl proteins in rat liver were detected by western blotting (*n* = 3). MW, molecular weight marker. Values are expressed as the mean ± SD (*n* = 6) for all groups. ^##^ *p* < 0.01 and ^#^ *p* < 0.05 denote statistically significant differences between the CON and MOD groups. ** *p* < 0.01 and * *p* < 0.05 denote statistically significant differences with MOD group.

ALT and AST are released after hepatocellular injury, making them useful markers for measuring liver function [26]. As shown in Figure 5a,b, the MOD group showed significantly increase (*p* < 0.05) in AST and ALT activities, while the reverse trend was observed for PD and TBP (*p* < 0.05). TBP effects on oxidative factors in hepatic tissues are presented in Figure 5c–g. In comparison with the CON group rats, as shown in the figure, the levels of CAT, SOD, GSH, and T-AOC in the liver tissue were reduced, and the levels of MDA were increased of HFD-fed rats (all *p* < 0.05). Intriguingly, PD could modulate the levels of SOD, GSH, T-AOC, and MDA (all *p* < 0.05) but had no effect on CAT levels, while the TBP could modulate GSH, T-AOC, and MDA levels (all *p* < 0.05) but had no effect on CAT and SOD levels.

Carbonylated proteins are considered to be indicators of oxidative damage, and in this research, the extent of carbonylation in rat liver was assessed by western blotting. As shown in Figure 5h, the oxidation level of liver protein carbonyls in each group can be seen. The molecular weight of rat liver protein carbonyls is mainly concentrated in 40~66 kDa. In contrast to the CON group, the MOD group obviously had extremely severe liver carbonyl oxidation. The liver carbonyl oxidation in the PD group was greatly improved, while the TBP group had a certain reduction trend. The above results demonstrated that TBP supplementation could improve hepatic dysfunction markers and antioxidant capacity of body.

### 3.5. Fecal Analysis

**Figure 6:**
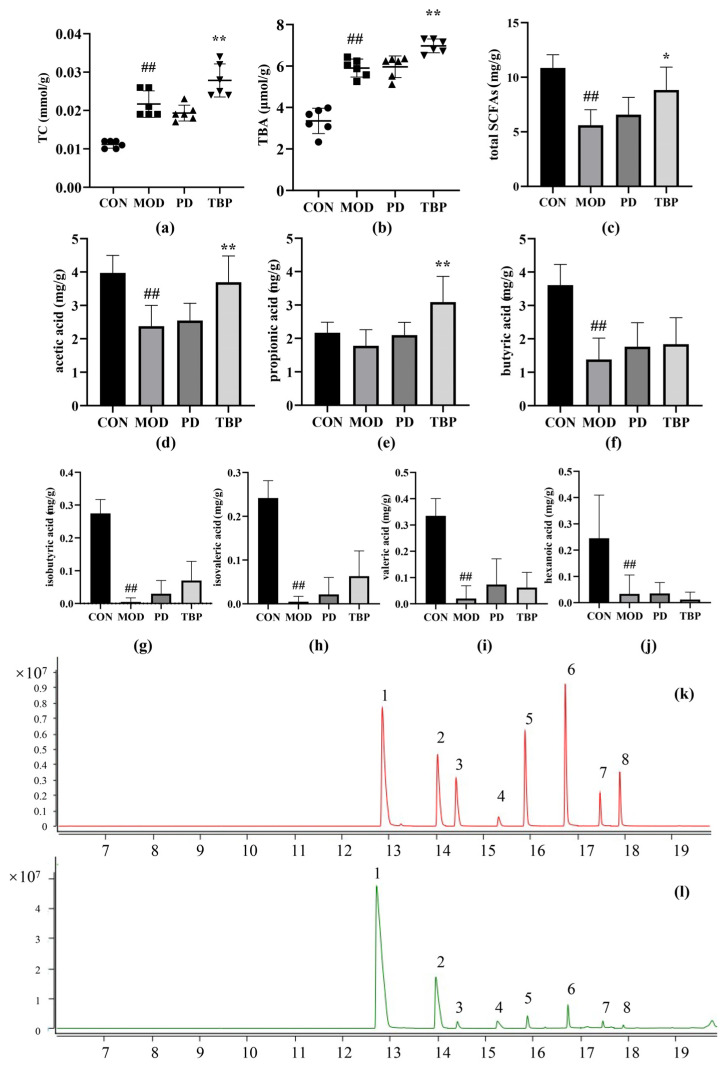
Effects of TBP administration on the fecal TC (**a**) and TBA (**b**) levels in SD rats fed on HFD. Effects of TBP on fecal short-chain fatty acid production: total SCFAs (**c**), acetic acid (**d**), propionic acid (**e**), butyric acid (**f**), isobutyric acid (**g**), isovaleric acid (**h**), valeric acid (**i**), and hexanoic acid (**j**). Chromatograms of short-chain fatty acid standard (**k**) and sample (**l**). 1: acetic acid; 2: propionic acid; 3: isobutyric acid; 4: butyric acid; 5: isovaleric acid; 6: valeric acid; 7: 4-methylvaleric acid (internal standard); 8: hexanoic acid. Values are expressed as the mean ± SD (*n* = 6) for all groups. ^##^ *p* < 0.01 denotes statistically significant differences between the CON and MOD groups. ** *p* < 0.01 and * *p* < 0.05 denote statistically significant differences with MOD group.

The liver catalyzes the oxidation of cholesterol through cholesterol 7-α hydroxylase to produce bile acids, which are therefore the major form of cholesterol excreted from the body. To assess whether the reduction in hepatic lipid accumulation by TBP intake in hyperlipidemic rats is caused by increased fecal cholesterol excretion, fecal biochemical indices, such as TC and TBA, were valuated. The test values are shown in Figure 6a,b, where the levels of TC and TBA in feces of rats from TBP group were dramatically higher than that from MOD group (*p* < 0.01). The increase in fecal bile acid excretion caused a decrease in bile acid levels in the intestinal and hepatic cycles, with a consequent increase conversion of cholesterol to bile acids in the liver [27]. Thus, intake of TBP may effectively reduce serum and hepatic lipid accumulation by promoting fecal lipid and TBA excretion in hyperlipidemic rats.

SCFAs are derived from intestinal flora fermenting carbohydrates and proteins in the colon that are not digested and absorbed by the host, with a high proportion of acetic acid, propionic acid, and butyric acid. As shown in Figure 6c–j, the content of total SCFAs, acetic acid, butyric acid, isobutyric acid, isovaleric acid, valeric acid, and hexanoic acid was significantly lower in the MOD group compared to the CON group (all *p* < 0.01). The contents of SCFAs (*p* < 0.05), acetic acid (*p* < 0.01) and propionic acid (*p* < 0.01) were substantially higher in the TBP group compared with the MOD group, while there was no obvious discrepancy in the PD group. The chromatograms of the short-chain fatty acid standard and the sample are shown in Figure 6k,l.

### 3.6. TBP Modulated Gut Microbiota in HFD Rats

**Figure 7:**
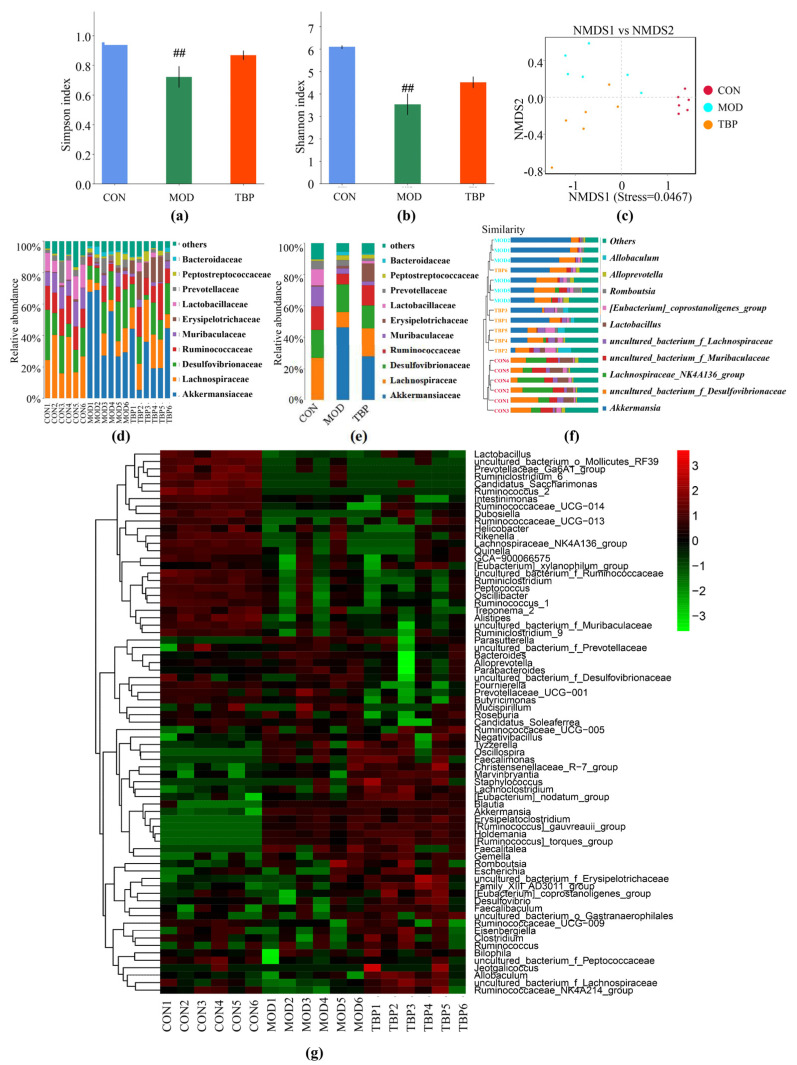
Effect of TBP on gut microbiota in the HFD-fed rats. (**a**) The alpha diversity of Simpson index. (**b**) The alpha diversity of Shannon index. (**c**) The beta diversity analysis by weighted unifrac distance-based NMDS. (**d**) Relative abundances of gut microbiota in each rat at the family levels. (**e**) Relative abundances of gut microbiota in each group at the family levels. (**f**) Clustering of the microbial communities based on weighed unifrac distance (at the genus level). (**g**) Heat map of gut microbiota (at the genus level). ^##^ *p* < 0.01 denotes statistically significant differences between the CON and MOD groups.

Gut microbiota is considered to be causally involved in the pathogenesis of lipid metabolism disorders. In this part, an investigation of how TBP regulates the cecum fecal microbiota of rats on a high-fat diet was made by using bacterial 16S rRNA high-throughput sequencing. Assessment of species abundance and evenness was done using Simpson’s and Shannon’s indices. The outcomes are displayed in Figure 7a,b. In cecum fecal samples, Simpson index and Shannon index showed a reduced diversity in the MOD group in comparison to the CON group (*p* < 0.01). TBP supplementation showed a beneficial result on bacterial alpha diversity in HFD-fed rats. The results showed that HFD and TBP supplementation could influence changes in gut microbiota. Beta diversity analysis by weighted unifrac distance-based non-metric multi-dimensional scaling (NMDS) on the basis of OTU abundance was undertaken to outline the extent of the similarities among the gut microbiota compositions after each treatment. NMDS demonstrated separate clustering of microbiota compositions in the cecum for each group rats (Figure 7c). Hierarchical clustering plots showed that the constituent of the HFD-induced gut microbiota was different from that of CON rats. Some changes in gut microbiota composition were observed after TBP supplementation, with a clear separation of clusters formed by TBP and MOD (Figure 7f). Thus, oral administration of TBP modestly altered the structure of the gut microbiota.

Taxonomic analysis showed a marked impact of TBP upon the gut microbiota. Figure 7d,e presents the relative abundance of the top 10 families from each group. Compared with the CON group, the relative abundance of Akkermansiaceae was substantially higher in the MOD group, while the relative abundance of Lachnospiraceae, Ruminococcaceae, Muribaculaceae, and Lactobacillaceae was substantially lower. TBP supplementation significantly increased the relative abundance of Lachnospiraceae, Ruminococcaceae, and Erysipelotrichaceae and decreased the relative abundance of Akkermansia in the intestine of HFD diet rats.

At the genus level (Figure 7f,g), compared with MOD group, TBP treatment showed a significant increase in *uncultured_bacterium_f_Lachnospiraceae* (family Lachnospiraceae), *Blautia* (family Lachnospiraceae), (*Eubacterium)_coprostanoligenes_group* (family Ruminococcaceae), and *Ruminococcaceae_NK4A214_group* (family Ruminococcaceae), while *Akkermansia* was significantly reduced.

### 3.7. Metabolomics Analysis

**Figure 8:**
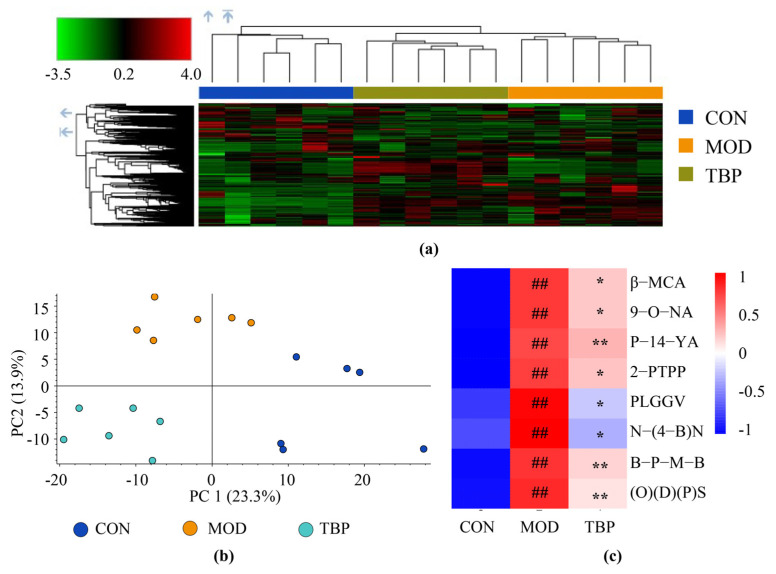
Effect of TBP on plasma metabolites in the HFD-fed rats. (**a**) Heat map of plasma metabolites. (**b**) PCA analysis of plasma metabolites. (**c**) Heat map of differential metabolites between groups: β-MCA, β-Muricholic acid; 9-O-NA, 9-{[(2E)-4-(3,4-Dihydroxy-5-{[3-(3-hydroxy-2-butanyl)-2-oxiranyl]methyl}tetrahydro-2H-pyran-2-yl)-3-methyl-2-butenoyl]oxy}nonanoic acid; P-14-YA, (3beta,6beta,14R)-3,5,6,10,16-Pentahydroxygrayanotoxan-14-yl acetate; 2-PTPP, 2-({[Dimethyl(2-methyl-2-propanyl)silyl]oxy}methyl)-5-pentyl-3,3a,11c,11d-tetrahydronaphtho[1′,2′:2,3]pentaleno[1,6-bc]pyran-4(2H)-one; PLGGV, Phe-Leu-Glu-Glu-Val; N-(4-B)N, N-(4-{(2S,4S,5R,6R)-4-{[Allyl(cyclopentyl)amino]methyl}-6-[4-(hydroxymethyl)phenyl]-5-methyl-1,3-dioxan-2-yl}benzyl)nicotinamide; B-P-M-B, 1,1-Bis[4-(benzyloxy)phenyl]-2-(4-methoxyphenyl)-2-butanol; (O)(D)(P)S, ({5-[2-(2,3-Dimethoxyphenyl)-2-propen-1-yl]-5-methyl-5,6,7,8-tetrahydro-2-naphthalenyl}oxy)(dimethyl)(2-methyl-2-propanyl)silane. Values are expressed as the mean (*n* = 6) for all groups. ^##^ *p* < 0.01 and denotes statistically significant differences between the CON and MOD groups. ** *p* < 0.01 and * *p* < 0.05 denote statistically significant differences with MOD group.

As shown by the results of clustered heat map and PCA (Figure 8a,b), plasma metabolites in the CON, MOD, and TBP groups showed a significant separation. Thus, the metabolism of HFD-fed rats was remarkably altered after five weeks of TBP supplementation. Among the metabolites with dramatical differences (*p* < 0.01) between the CON and MOD groups, eight metabolites that were back-regulated (*p* < 0.05) after TBP supplementation were found and identified (Figure 8c).

## 4. Discussion

Excessive consumption of high-fat and sugar by the individual may lead to disorders of lipid metabolism, such as hyperlipidemia, atherosclerosis, and nonalcoholic fatty liver. Serum TC, TG, LDL-C, and HDL-C levels are the main indicators that can reflect the body’s lipid metabolism. Abnormal increases in blood lipid levels can lead to disease-threatening conditions. A hyperlipidemic rat model was established in our laboratory by HFD. The results showed that serum TC, TG, and LDL-C levels were markedly raised, and HDL-C levels were dramatically reduced in hyperlipidemic rats. A long-term HFD may also lead to significant weight gain, an increased liver index, and an increased abdominal lipid rate. Our results demonstrate that the HFD-induced hyperlipidemic rat model is successful. After administration of TBP, serum LDL-C levels were markedly diminished, HDL-C levels were markedly raised, and liver index and abdominal lipid rate were dramatically decreased in hyperlipidemic rats, suggesting that TBP regulates lipid metabolism within a certain range. In addition, TBP decreased the liver steatosis markers TC, TG, LDL-C, and FFA, indicating that TBP could restore HFD-induced fatty liver. For oxidative damage induced by hyperlipidemia, lipid oxidation levels were elevated in rats on HFD [28]. Supplementation of TBP increased the hepatic antioxidant properties and attenuated hepatic protein carbonylation in hyperlipidemic rats. Furthermore, TBP also reduces serum and liver lipid accumulation by promoting fecal cholesterol and TBA excretion.

Due to its low digestibility, TBP has functions similar to those associated with resistant starch or dietary fiber. Dietary TBP can evade stomach and small intestine digestion and promote the proliferation of helpful bacteria through fermentation, improving gut microbial diversity and producing beneficial metabolites, most notably SCFAs. SCFAs, including acetic acid, propionic acid, and butyric acid, are the main final outputs of cecal and colonic fermentation, which are associated with energy metabolism. There is substantial evidence that SCFAs are effective in alleviating diet-induced hyperlipidemia, particularly hypercholesterolemia [29]. Actually, SCFAs have been shown to reduce serum and hepatic lipid levels by via reducing cholesterol and fatty acid synthesis substrates (e.g., acetyl-CoA), inhibiting genes that regulate these substrates (e.g., fatty acid synthase), or facilitating fecal bile acid excretion [30,31,32,33]. In this study, supplementation with TBP contributed to increase the level of SCFAs, acetic acid, and propionic acid. Thus, dietary TBP diminished serum and liver lipid levels in rats, possibly by promoting the production of SCFAs and hence increasing fecal bile acid excretion. However, whether there is an effect on lipid metabolism-related genes expression still needs to be further determined.

A growing amount of evidence for the critical role of the gut microbiota in the lipid metabolism disorders. The composition of the gut microbiota is reshaped in obese individuals, which is characterized by dysfunctional biology and reduced microbiota diversity. In the present study, the cecum microbiota of rats was analyzed, and the HFD diet reduced its species richness and species diversity, which was somewhat improved by TBP supplementation. Besides, TBP supplementation modulated the cecum microbiota of HFD diet rats by lowering the relative abundance of Ackermannia and raising the relative abundance of Lachnospiraceae and the Ruminococcaceae, which is consistent with the study of fucoidan from *Laminaria japonica* [34].

The richness of Akkermansia in the intestine is about 1% to 4% [33]. The anti-obesity effect of Akkermansia in humans and animals has been reported in the literature [35,36]. However, the effect of Akkermansia on the body is perhaps not always positive. It has been published that Akkermansia can cause intestinal inflammation via engulfing the mucin layer of mice on a long-term fiber-free diet [37]. In addition, a HFD predisposes mice to an increased risk of colorectal cancer, with a substantially higher proportion of Akkermansia bacteria in their colonic microbiota [37,38], and exhibits pro-inflammatory properties [39,40]. Additionally, there are reports that inflammatory bowel disease and type 2 diabetes are also related to more abundant Akkermansia, which can be reversed by eating seaweed extract [41,42,43]. In our study, we found that HFD was beneficial to the emergence of the Akkermensia (abundance of 46%), a finding consistent with similar observations in rat models [44,45]. Whereas the drastically increased relative abundance of Akkermansia due to HFD was dampened by TBP supplementation. The inhibitory effect of TBP supplementation may be attributed to the indigestible nature. A similar finding also indicated that bamboo shoot fiber intake inhibited the relative abundance of Akkermansia [46]. Therefore, these results indicate that the role of Akkermansia in obesity, type 2 diabetes, hyperlipidemia, and other metabolic diseases should be further studied.

The Lachnospiraceae and the Ruminococcaceae are principal families of intestinal bacteria engaged in carbohydrate metabolisms. They include, for example, the major butyrate-producing species, species that transform lactate into butyrate or propionate, as well as species that undergo reductive acetogenesis [47]. Besides, the population size of Ruminococcaceae is strongly and negatively linked to alcoholic cirrhotics, hepatic encephalopathy, and non-alcoholic fatty liver disease [34]. *Blautia*, a beneficial bacterium in the gut [48], inhibits insulin signaling and fat accumulation by the activation of G protein-coupled receptors GPR41 and GPR43, attenuating the diseases associated with obesity [49]. Li et al. [50] found that the *[Eubacterium]_coprostanoligenes_group* converted intestinal cholesterol to coprostanol in hens. Those results indicate that TBP enhances the abundance of SCFA producers, facilitating its lipid-regulating effect. In conclusion, our results indicated that TBP can modulate the richness of gut microbiota.

Among the candidate plasma differential metabolites, β-Muricholic acid (β-MCA) has attracted attention. β-MCA is a murine specific bile acid that is usually produced in the liver by conversion of goose deoxycholic acid. In addition, β-MCA is usually combined with taurine in the liver to form T-β-MCA, which is a potent antagonist of the farnesoid X receptor [51]. Farnesoid X receptor is broadly distributed in the liver, intestine, and other organs and is mainly involved in regulating bile acid metabolism and cholesterol homeostasis [52,53]. In this experiment, a significant upregulation of free β-Muricholic acid was observed in the plasma metabolites of the MOD group, and it was significantly downregulated after TBP supplementation. Therefore, it is speculated that TBP supplementation may have an effect on bile acid metabolism in rats on HFD. In future studies, the effect of TBP on the metabolism of hepatic and intestinal bile acids in HFD rats can be studied by targeted metabolomics to analyze the metabolic pathways.

## 5. Conclusions

In conclusion, TBP could play an important role in modifying the lipid profile and decreasing lipid peroxidation in rats on HFD. The potential mechanism of action is, on the one hand, to increase the abundance of the Lachnospiraceae and the Ruminococcaceae by modulating the gut microbiota, facilitating the generation of short-chain fatty acids, and increasing fecal bile acid excretion and, on the other hand, may be related to the improvement of bile acid metabolism.

## Data Availability

Not applicable.

## References

[1] Kim S., Hong J., Jeon R., Kim H.S. (2016). Adzuki Bean Ameliorates Hepatic Lipogenesis and Proinflammatory Mediator Expression in Mice Fed a High-Cholesterol and High-Fat Diet to Induce Nonalcoholic Fatty Liver Disease. Nutr. Res..

[2] Venkatakrishnan K., Chiu H.F., Wang C.K. (2019). Extensive Review of Popular Functional Foods and Nutraceuticals against Obesity and Its Related Complications with a Special Focus on Randomized Clinical Trials. Food Funct..

[3] Li W., Zhang K., Yang H. (2018). Pectin Alleviates High Fat (Lard) Diet-Induced Nonalcoholic Fatty Liver Disease in Mice: Possible Role of Short-Chain Fatty Acids and Gut Microbiota Regulated by Pectin. J. Agric. Food. Chem..

[4] Nie X., Chen Z., Pang L., Wang L., Jiang H., Chen Y., Zhang Z., Fu C., Ren B., Zhang J. (2020). Oral Nano Drug Delivery Systems for the Treatment of Type 2 Diabetes Mellitus: An Available Administration Strategy for Antidiabetic Phytocompounds. Int. J. Nanomed..

[5] Davignon J., Montigny M., Dufour R. (1992). Hmg-Coa Reductase Inhibitors: A Look Back and a Look Ahead. Can. J. Cardiol..

[6] Sarin S., Kaman L., Dahiya D., Behera A., Medhi B., Chawla Y. (2016). Effects of Preoperative Statin on Liver Reperfusion Injury in Major Hepatic Resection: A Pilot Study. Updates Surg..

[7] Broeders N., Knoop C., Abramowicz D. (1999). Drug Treatment of Lipid Disorders. N. Engl. J. Med..

[8] Wu D.T., He Y., Fu M.X., Gan R.Y., Hu Y.C., Peng L.X., Zhao G., Zou L. (2022). Structural Characteristics and Biological Activities of a Pectic-Polysaccharide from Okra Affected by Ultrasound Assisted Metal-Free Fenton Reaction. Food Hydrocoll..

[9] Ahmed A., Khalid N., Ahmad A., Abbasi N.A., Latif M.S.Z., Randhawa M.A. (2014). Phytochemicals and Biofunctional Properties of Buckwheat: A Review. J. Agric. Sci..

[10] Fabjan N., Rode J., Kosir I.J., Wang Z.I. (2003). Tartary Buckwheat (*Fagopyrum tataricum* Gaertn.) as a Source of Dietary Rutin and Quercitrin. J. Agric. Food Chem..

[11] Zou L., Wu D., Ren G., Hu Y., Peng L., Zhao J., Garcia-Perez P., Carpena M., Prieto M.A., Cao H. (2021). Bioactive Compounds, Health Benefits, and Industrial Applications of Tartary Buckwheat (*Fagopyrum tataricum*). Crit. Rev. Food Sci. Nutr..

[12] Kayashita J., Shimaoka I., Nakajoh M., Yamazaki M., Kato N. (1997). Consumption of Buckwheat Protein Lowers Plasma Cholesterol and Raises Fecal Neutral Sterols in Cholesterol-Fed Rats Because of Its Low Digestibility. J. Nutr..

[13] Zhang C., Rui Z., Li Y.M., Ning L. (2017). Plasma Cholesterol-Lowering Activity of Tartary Buckwheat Protein. J. Agric. Food Chem..

[14] Zhou X.L., Yan B.B., Xiao Y., Zhou Y.M., Liu T.Y. (2018). Tartary Buckwheat Protein Prevented Dyslipidemia in High-Fat Diet-Fed Mice Associated with Gut Microbiota Changes. Food Chem. Toxicol..

[15] Chen Y.F., Jin L., Li Y.H., Xia G.B., Chen C., Zhang Y. (2018). Bamboo-Shaving Polysaccharide Protects against High-Diet Induced Obesity and Modulates the Gut Microbiota of Mice. J. Funct. Foods.

[16] Hul M.V., Karnik K., Canene-Adams K., Souza M.D. (2020). Comparison of the Effects of Soluble Corn Fiber and Fructooligosaccharides on Metabolism, Inflammation and Gut Microbiome of High-Fat Diet Fed Mice. J. Nutr..

[17] Anhê F.F., Varin T.V., Barz M.L., Pilon G., Dudonné S., Trottier J., St-Pierre P., Harris C.S., Lucas M. (2018). Arctic Berry Extracts Target the Gut–Liver Axis to Alleviate Metabolic Endotoxaemia, Insulin Resistance and Hepatic Steatosis in Diet-Induced Obese Mice. Diabetologia.

[18] Liu H.H., Tian R., Wang H., Feng S.Q., Li H.Y., Xiao Y., Luan X.D., Zhang Z.Y., Shi N., Niu H.T. (2020). Gut Microbiota from Coronary Artery Disease Patients Contributes to Vascular Dysfunction in Mice by Regulating Bile Acid Metabolism and Immune Activation. J. Transl. Med..

[19] Wang T.J., Larson M.G., Vasan R.S., Cheng S., Rhee E.P., McCabe E., Lewis G.D., Fox C.S., Jacques P.F., Fernandez C. (2011). Metabolite Profiles and the Risk of Developing Diabetes. Nat. Med..

[20] Guo X., Zhu K., Zhang H., Yao H. (2007). Purification and Characterization of the Antitumor Protein from Chinese Tartary Buckwheat (*Fagopyrum tataricum* Gaertn.) Water-Soluble Extracts. J. Agric. Food Chem..

[21] Zhou Y., Jiang Y., Shi R., Chen Z., Li Z., Wei Y., Zhou X. (2020). Structural and Antioxidant Analysis of Tartary Buckwheat (*Fagopyrum tartaricum* Gaertn.) 13s Globulin. J. Sci. Food Agric..

[22] Colombo G., Clerici M., Garavaglia M.E., Giustarini D., Rossi R., Milzani A., Dalle-Donne I. (2016). A Step-by-Step Protocol for Assaying Protein Carbonylation in Biological Samples. J. Chromatogr. B Analyt. Technol. Biomed Life Sci..

[23] Peng L.X., Zhang Q., Zhang Y.H., Yao Z.D., Song P.P., Wei L.J., Zhao G., Yan Z.Y. (2020). Effect of Tartary Buckwheat, Rutin, and Quercetin on Lipid Metabolism in Rats during High Dietary Fat Intake. Food Sci. Nutr..

[24] Cesare L., Rubert J., Fava F., Tuohy K., Mattivi F., Vrhovsek U. (2017). Development of a Fast and Cost-Effective Gas Chromatography—Mass Spectrometry Method for the Quantification of Short-Chain and Medium-Chain Fatty Acids in Human Biofluids. Anal. Bioanal. Chem..

[25] Wang J., Xiao J., Liu X., Geng F., Huang Q., Zhao J., Xiang D., Zhao G. (2019). Analysis of Tartary Buckwheat (*Fagopyrum tataricum*) Seed Proteome Using Offline Two-Dimensional Liquid Chromatography and Tandem Mass Spectrometry. J. Food Biochem..

[26] Loeb W.F., Quimby F.W. (1989). The Clinical Chemistry of Laboratory Animals.

[27] Maria A., Ellegård L., Andersson H. (2002). Oat Bran Stimulates Bile Acid Synthesis within 8 H as Measured by 7α-Hydroxy-4-Cholesten-3-One. Am. J. Clin. Nutr..

[28] Cao Y.N., Zou L., Li W., Song Y., Zhao G., Hu Y.C. (2020). Dietary Quinoa (*Chenopodium quinoa* Willd.) Polysaccharides Ameliorate High-Fat Diet-Induced Hyperlipidemia and Modulate Gut Microbiota. Int. J. Biol. Macromol..

[29] Hara H., Haga S., Aoyama Y., Kiriyama S. (1999). Short-Chain Fatty Acids Suppress Cholesterol Synthesis in Rat Liver and Intestine. J. Nutr..

[30] Fushimi T., Suruga K., Oshima Y., Fukiharu M., Tsukamoto Y., Goda T. (2006). Dietary Acetic Acid Reduces Serum Cholesterol and Triacylglycerols in Rats Fed a Cholesterol-Rich Diet. Br. J. Nutr..

[31] Nguyen T.D., Prykhodko O., Hallenius F.F., Nyman M. (2019). Monobutyrin Reduces Liver Cholesterol and Improves Intestinal Barrier Function in Rats Fed High-Fat Diets. Nutrients.

[32] Tolhurst G., Heffron H., Lam Y.S., Parker H.E., Habib A.M., Diakogiannaki E., Cameron J., Grosse J., Reimann F., Gribble F.M. (2012). Short-Chain Fatty Acids Stimulate Glucagon-Like Peptide-1 Secretion Via the G-Protein-Coupled Receptor Ffar2. Diabetes.

[33] Brass E.P., Beyerinck R.A. (1988). Effects of Propionate and Carnitine on the Hepatic Oxidation of Short- and Medium-Chain-Length Fatty Acids. Biochem. J..

[34] Shang Q.S., Shan X.D., Cai C., Hao J.J., Li G.Y., Yu G.L. (2016). Dietary Fucoidan Modulates the Gut Microbiota in Mice by Increasing the Abundance of Lactobacillus and Ruminococcaceae. Food Funct..

[35] Depommier C., Everard A., Druart C., Plovier H., van Hul M., Vieira-Silva S., Falony G., Raes J., Maiter D., Delzenne N.M. (2019). Supplementation with Akkermansia Muciniphila in Overweight and Obese Human Volunteers: A Proof-of-Concept Exploratory Study. Nat. Med..

[36] Ussar S., Griffin N.W., Bezy O., Fujisaka S., Vienberg S., Softic S., Deng L.X., Bry L., Gordon J.I., Kahn C.R. (2016). Interactions between Gut Microbiota, Host Genetics and Diet Modulate the Predisposition to Obesity and Metabolic Syndrome (Vol 22, Pg 516, 2015). Cell Metab..

[37] Desai M.S., Seekatz A.M., Koropatkin N.M., Kamada N., Hickey C.A., Wolter M., Pudlo N.A., Kitamoto S., Terrapon N., Muller A. (2016). A Dietary Fiber-Deprived Gut Microbiota Degrades the Colonic Mucus Barrier and Enhances Pathogen Susceptibility. Cell.

[38] Wang C.Z., Huang W.H., Zhang C.F., Wan J.Y., Wang Y., Yu C., Williams S., He T.C., Du W., Musch M.W. (2018). Role of Intestinal Microbiome in American Ginseng-Mediated Colon Cancer Protection in High Fat Diet-Fed Aom/Dss Mice. Clin. Transl. Oncol..

[39] Wang H., Guan L.N., Li J., Lai M.D., Wen X.D. (2018). The Effects of Berberine on the Gut Microbiota in Apc (Min/+) Mice Fed with a High Fat Diet. Molecules.

[40] Wang C.Z., Yu C.H., Wen X.D., Chen L.N., Zhang C.F., Calway T., Qiu Y.P., Wang Y.W., Zhang Z.Y., Anderson S. (2016). American Ginseng Attenuates Colitis-Associated Colon Carcinogenesis in Mice: Impact on Gut Microbiota and Metabolomics. Cancer Prev. Res..

[41] Seregin S.S., Golovchenko N., Schaf B., Chen J.C., Pudlo N.A., Mitchell J., Baxter N.T., Zhao L.L., Schloss P.D., Martens E.C. (2017). Nlrp6 Protects Il10(-/-) Mice from Colitis by Limiting Colonization of Akkermansia Muciniphila (Vol 19, Pg 733, 2017). Cell Rep..

[42] Qin J.J., Li Y.R., Cai Z.M., Li S.H., Zhu J.F., Zhang F., Liang S.S., Zhang W.W., Guan Y.L., Shen D.Q. (2012). A Metagenome-Wide Association Study of Gut Microbiota in Type 2 Diabetes. Nature.

[43] Yan X., Yang C.F., Lin G.P., Chen Y.Q., Miao S., Liu B., Zhao C. (2019). Antidiabetic Potential of Green Seaweed Enteromorpha Prolifera Flavonoids Regulating Insulin Signaling Pathway and Gut Microbiota in Type 2 Diabetic Mice. J. Food Sci..

[44] Hamilton M.K., Boudry G., Lemay D.G., Raybould H.E. (2015). Changes in Intestinal Barrier Function and Gut Microbiota in High-Fat Diet-Fed Rats Are Dynamic and Region Dependent. Am. J. Physiol.-Gastrointest. Liver Physiol..

[45] Fak F., Jakobsdottir G., Kulcinskaja E., Marungruang N., Matziouridou C., Nilsson U., Stalbrand H., Nyman M. (2015). The Physico-Chemical Properties of Dietary Fibre Determine Metabolic Responses, Short-Chain Fatty Acid Profiles and Gut Microbiota Composition in Rats Fed Low- and High-Fat Diets. PLoS ONE.

[46] Li X.F., Guo J., Ji K.L., Zhang P. (2016). Bamboo Shoot Fiber Prevents Obesity in Mice by Modulating the Gut Microbiota. Sci. Rep..

[47] Flint H.J., Scott K.P., Duncan S.H., Louis P., Forano E. (2012). Microbial Degradation of Complex Carbohydrates in the Gut. Gut Microbes.

[48] Liu X., Mao B., Gu J., Wu J., Cui S., Wang G., Zhao J., Zhang H., Chen W. (2021). Blautia—A New Functional Genus with Potential Probiotic Properties?. Gut Microbes.

[49] Kimura I., Ozawa K., Inoue D., Imamura T., Kimura K., Maeda T., Terasawa K., Kashihara D., Hirano K., Tani T. (2013). The Gut Microbiota Suppresses Insulin-Mediated Fat Accumulation via the Short-Chain Fatty Acid Receptor Gpr43. Nat. Commun..

[50] Li L., Baumann C.A., Meling D.D., Sell J.L., Beitz D.C. (1996). Effect of Orally Administered Eubacterium Coprostanoligenes Atcc 51222 on Plasma Cholesterol Concentration in Laying Hens. Poult. Sci..

[51] Martinot E., Sedes L., Baptissart M., Lobaccaro J.M., Caira F., Beaudoin C., Volle D.H. (2017). Bile Acids and Their Receptors. Mol. Asp. Med..

[52] Gonzalez F.J., Jiang C.T., Xie C., Patterson A.D. (2017). Intestinal Farnesoid X Receptor Signaling Modulates Metabolic Disease. Dig. Dis..

[53] Preidis G.A., Kim K.H., Moore D.D. (2017). Nutrient-Sensing Nuclear Receptors Ppar Alpha and Fxr Control Liver Energy Balance. J. Clin. Investig..

